# Does Cannabis Extract Obtained From Cannabis Flowers With Maximum Allowed Residual Level of Aflatoxins and Ochratoxin a Have an Impact on Human Safety and Health?

**DOI:** 10.3389/fmed.2021.759856

**Published:** 2021-11-15

**Authors:** Tijana Serafimovska, Sasho Stefanovski, Joachim Erler, Zlatko Keskovski, Gjoshe Stefkov, Marija Mitevska, Marija Darkovska Serafimovska, Trajan Balkanov, Jasmina Tonic Ribarska

**Affiliations:** ^1^Faculty of Pharmacy, University Ss. Cyril and Methodius, Skopje, North Macedonia; ^2^NYSK Holdings, Company for Growing, Extraction and Producing of Pharmaceutical Dosage Forms of Medical Cannabis, Skopje, North Macedonia; ^3^Diapharm GmbH & Co. KG, Münster, Germany; ^4^Faculty of Medical Sciences, University Goce Delcev, Shtip, North Macedonia; ^5^Department of Pharmacology and Toxicology, Faculty of Medicine, University Ss. Cyril and Methodius, Skopje, North Macedonia

**Keywords:** mycotoxins, aflatoxins, ochratoxin A, determination liquid chromatography with tandem mass spectrometry (LC/MS/MS), cannabis extracts

## Abstract

**Aim:** The aim of this study was to investigate whether the cannabis extract obtained from cannabis flowers that contain the maximum allowed level of mycotoxins affects human safety and health. For that purpose, a novel liquid chromatography with tandem mass spectrometry (LC/MS/MS) method was developed and validated for the determination of aflatoxins and ochratoxin A (OchA) in cannabis extracts to demonstrate that this analytical method is suitable for the intended experimental design.

**Methods:** Experimental design was done by adding maximum allowed concentration of aflatoxins (B1, B2, G1, G2) and OchA according to the European Pharmacopeia related to cannabis flowers. The concentration of aflatoxins and OchA was determined using the same LC/MS/MS analytical method in the starting material (dry flower) before preparing the spiked sample and after obtaining decarboxylated extract with ethanol 96%.

**Results:** The results obtained indicate that aflatoxins and OchA, primarily added to the cannabis dried flowers, were also determined into the obtained final extract in amounts much higher (m/m) than in the starting plant material.

**Conclusion:** With this experiment, we have shown that mycotoxins, especially aflatoxins, which are extremely toxic secondary metabolites, can reach critical values in cannabis extracts obtained from dry cannabis flowers with the maximum allowed quantity of mycotoxins. This can pose a great risk to consumers and their health especially to those with compromised immune systems.

## Introduction

Medicinal products based on *Cannabis sativa L*. (Cannabaceae) in traditional medicine, have been used for thousands of years in the treatment of various diseases (1). Although, there is a lack of evidence-based medical information that can prove the potential benefit of the therapy with medicinal cannabis preparations, recently, an increasing number of pharmacists have issued cannabis preparations to individual patients prescribed by their physicians (2).

To obtain cannabis-based preparations, standardized concentrated cannabinoid extracts, produced by a suitable extraction process of cannabinoids from cannabis flowers are used (2). The safety of the flowers as a starting material, in this case, is the most significant for human safety and health. Since there is no monograph in the European Pharmacopeia (Ph.Eur.) for quality testing of cannabis flowers, currently a revised monograph for cannabis flower (cannabis floss), published in the German Pharmacopeia 2018 (3), by the Federal Institute for Drugs and Medical Devices (BfArM) has instructed the obligatory procedure for quality testing of cannabis flowers in the European Union (4). However, a variety of herbal monographs are listed under the general monographs in Ph.Eur.: herbal drugs, herbal drug extracts, and herbal drug preparations. These general monographs are created to cover products and quality parameters, which are not mentioned in the individual monographs. Therefore, it is necessary to apply the individual monograph always in combination with these quality requirements (5).

According to the Guideline on specifications: test procedures and acceptance criteria for herbal substances (6), mycotoxins [aflatoxins, ochratoxin A (OchA)] are considered as impurities (contaminants) that can occur in the final extracts from starting materials (herbal drugs). In reference to this, Ph.Eur. gives the maximum allowed limits of these impurities (aflatoxins, as per Ph.Eur. 2.8.18 and OchA, as per Ph.Eur. 2.8.22) in herbal drugs.

### Impact of Mycotoxins on Human Safety and Health

Mycotoxins (aflatoxins and OchA) are secondary toxic metabolites, obtained primarily from fungal species (*Penicillium* and *Aspergillus*). Fungi and their metabolites contaminate the raw materials usually used in the preparation of products for human use (7). The presence of these contaminants in products for human use can cause various acute and chronic effects on human safety and health (8).

**Figure 1:**
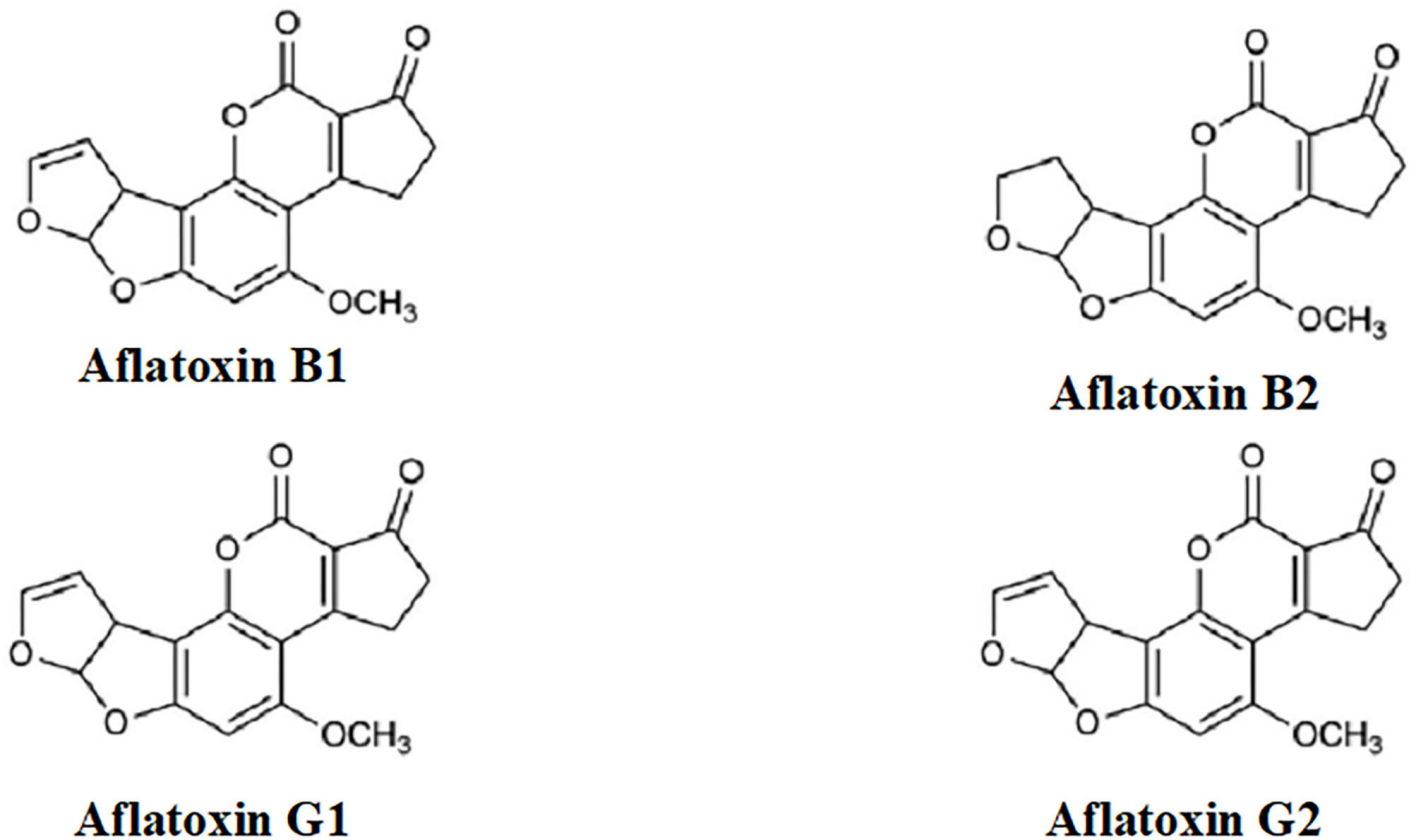
Structures of aflatoxins.

Aflatoxins (Figure 1) are extremely toxic secondary metabolites. According to their chemical structures, they are generally categorized into two groups: the difurocoumarocyclopentenone group (aflatoxin B1 and B2) and the difurocoumarolactone group (aflatoxin G1 and G2). The most toxic, carcinogenic, and mutagenic among all the aflatoxins is aflatoxin B1 (AfB1). Humans are mostly infected by direct ingestion (consuming) of infected herbal drugs, food, or herbal preparations (9).

**Figure 2:**
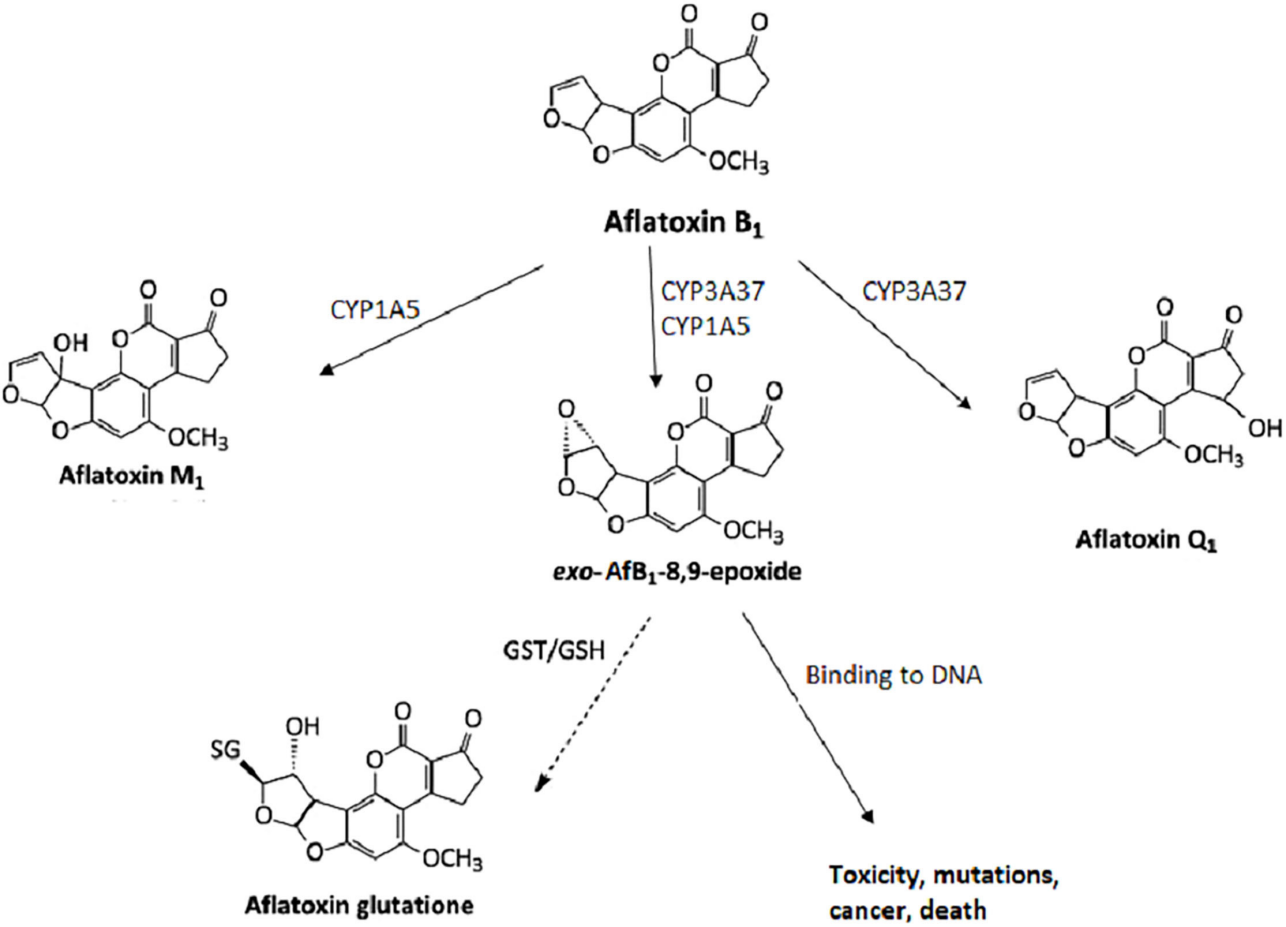
Metabolism of aflatoxin B1 in hepatocytes.

Mycotoxins are metabolized in the liver, and microorganisms in the digestive tract (10, 11). AfB1 itself is not carcinogenic, but in the human body, it is metabolized into carcinogenic metabolites. All enzymes necessary for the bioactivation of AfB1 are present in the nuclear envelope of the hepatocytes. The mono-oxygenase system in microsomes can transform the AfB1 into aflatoxin M1 (AfM1), aflatoxin Q1 (AfQ1), and exo-AfB1-8,9-epoxide (Figure 2) which can bind to deoxyribonucleic acid (DNA) and causes cytotoxicity, DNA damage, chromosomal anomalies, gene mutation, and cell transformation by attacking the nucleophilic hetero-atoms such as oxygen and nitrogen in the organic bases of nucleic acids and forming a strong covalent bond to the DNA (9).

Specific effects of Aflatoxins on human safety and health can be classified into two groups such as chronic toxicity and acute toxicity.

Carcinogenicity, hepatotoxicity, nephrotoxicity, and endocrine disorders have been related to chronic exposure to low levels of mycotoxins (12, 13). In some cases, allergic reactions, immune diseases, reproductive deficiencies, fetal alterations, and death, have also been related to chronic exposure to mycotoxins (14). The health risks associated with mycotoxin exposure arise from their toxicity and depend on the type of toxin, its metabolism, the immune system, and the health status of the exposed individual (15). Due to the carcinogenic risk associated with the mycotoxins, the International Agency for Research on Cancer (IARC) has evaluated and classified mycotoxins as carcinogenic to humans (Group 1), possibly carcinogenic to humans (Group 2B), or not classifiable as to its carcinogenicity to humans (Group 3), based on sufficient experimental data (16).

The main target organ for toxicity, mutagenicity, and carcinogenicity is the liver (17).

**Figure 3:**
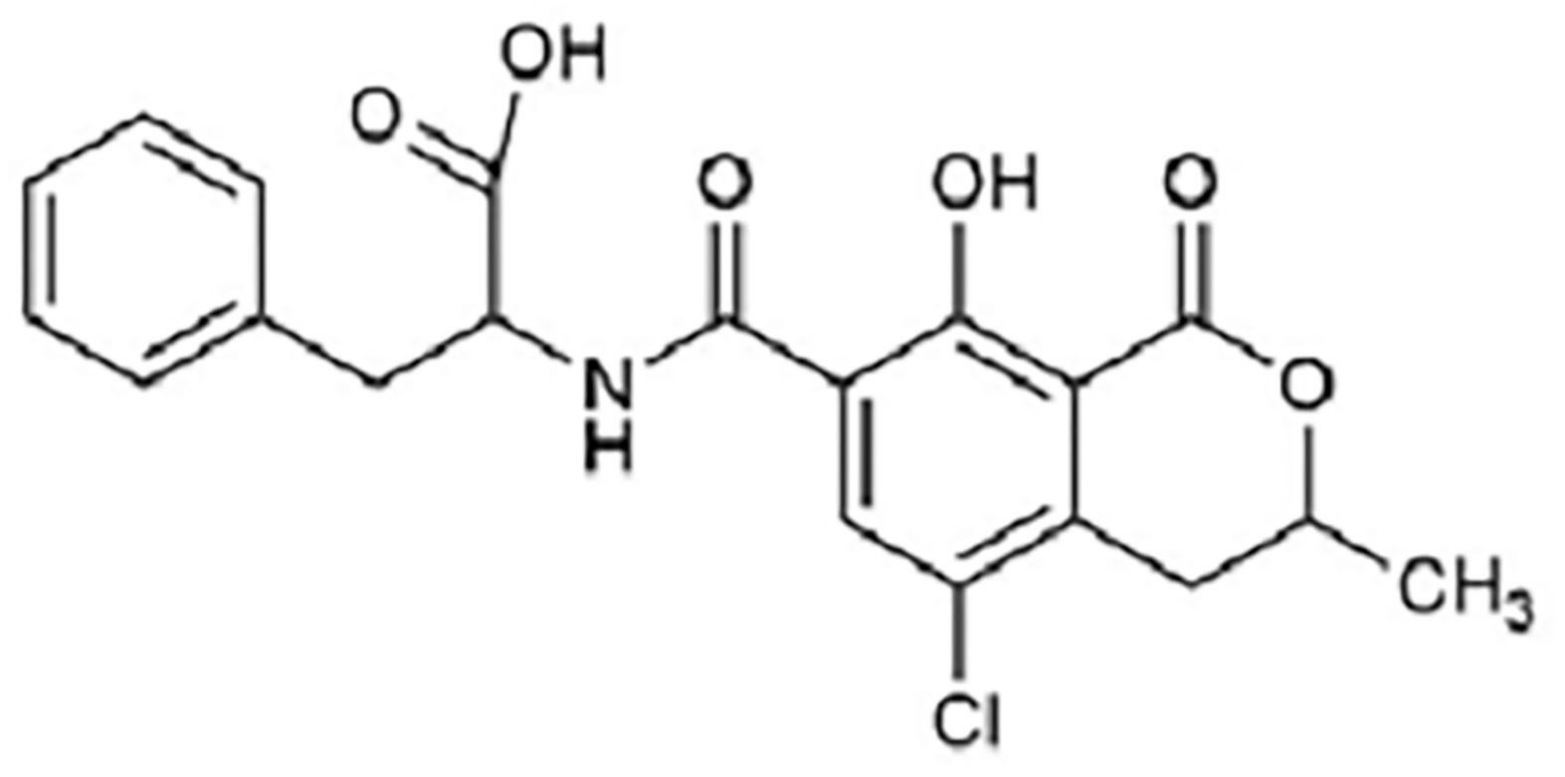
Structure of Ochratoxin A.

Ochratoxin A (Figure 3) is a powerful mycotoxin, responsible for chronic toxicity, such as nephrotoxicity, hepatotoxicity, teratogenicity, and immunotoxicity to humans (18). There are several *in vivo* and *in vitro* studies published regarding nephrotoxicity and hepatotoxicity of OchA, due to diverse metabolites of OchA, but the exact mechanism of toxicity of this mycotoxin is still unclear (19, 20).

Therefore, the aim of this study was to investigate whether the cannabis extract obtained from cannabis flowers that contain the maximum allowed level of mycotoxins affects human safety and health. There are many recently published manuscripts related to quantification techniques for the determination of mycotoxins in cannabis flower and cannabis extracts, inapplicable for the equipment we used (21, 22). For that reason, a novel LC/MS/MS method was developed and validated for the determination of aflatoxins and OchA in cannabis extracts to demonstrate that this analytical method is suitable for the intended experimental design, thus, achieving the set goal.

## Materials and Methods

### Chemicals and Regents

Liquid standards of AfB1 Cat.No.TSL-104-10, aflatoxin B2 (AfB2) Cat.No.TSL-105-10, aflatoxin G1 (AfG1) Cat.No.TSL-106-10, aflatoxin G2 (AfG2) Cat.No.TSL-107-10, and OchA Cat.No.TSL-504-5 were supplied by R-Biopharm (Germany). Other chemicals and reagents used in this study were LC/MS grade provided by Fisher Chemicals (UK).

Immunoaffinity columns (IAC) were obtained from R-Biopharm (Germany), Cat. No. RBRP112B.

### Apparatus

Liquid chromatography was performed on LC/MS/MS system (LC - 30AD series) equipped with a binary pump, vacuum degasser, standard autosampler, column compartment, and MS/MS detector (8045 series) from Shimadzu (Japan).

### Chromatographic Conditions

The separation was performed using a gradient method and reversed-phase Raptor Biphenyl LC column (100 x 2.1 mm, particle size 2.7 μm, Cat.No.980-18088) that provide the best separation characteristics, coupled with Raptor Biphenyl EXP guard column cartridge (5 x 2.1 mm, particle size 2.7 μm, cat.No.9309A0252) from Restek (USA). The choice of solvent for the preparation of standard solutions and extraction of mycotoxins (aflatoxins and OchA) from cannabis floss and extracts was made to eliminate the isobaric interferences resulting in spiked samples. It was determined that an extraction solvent of 50:50 = water: methanol with 0.1% formic acid results in samples (extracts) with removed isobaric interferences.

The mobile phase contains a mixture of 5 mM ammonium formate in water with 0.1% formic acid and 5 mM ammonium formate in methanol with 0.1% formic acid and a flow rate of 0.45 ml/min gave the best results. Analytical conditions for the gradient method are given in Table 1. The injection volume of samples was 10 μl.

**Table 1:** Analytical conditions for analysis of aflatoxins and ochratoxin A (gradient method).

**Column**	Raptor Biphenyl 100 mm × 2.1 mm, particle size 2.7 μm, (Cat.No.980-18088)		
**Guard column**	Raptor Biphenyl EXP Guard Column Cartridge 2.7 μm, 5 × 2.1 mm (cat.# 9309A0252)		
**Mobile phase A**	5 mM ammonium formate in water with 0.1% formic acid		
**Mobile phase B**	5 mM ammonium formate in methanol with 0.1% formic acid		
**Time program**	**Time (min.)**	**Flow (mL/min.)**	**%B**
	2.20	0.45	30
	2.40	0.45	50
	8.20	0.45	70
	11.20	0.45	75
	12.20	0.45	90
	12.60	0.45	90
	12.61	0.45	75
	13.20	0.45	75
	13.21	0.45	30
	16	0.45	30
**Oven temp**.	40°C		
**Sample temp**.	15°C		
**Inj. volume**	10 μL		
**MS/MS**	Shimadzu LCMS-8045		
**Ion mode**	ESI+		

Water: methanol = 50:50 (V/V) with 0.1% formic acid was used for the preparation of standard solutions and extraction of mycotoxins (aflatoxins and OchA) from cannabis flowers and extracts.

Chromatographic data were analyzed using LabSolution software from Shimadzu (Japan), following the requirements for chromatographic analysis.

Analyte transitions are given in Table 2.

**Table 2:** Analyte transitions.

**Analyte**	**Precursor Ion**	**Product Ion (Quantifier)**	**Product Ion (Qualifier)**
Aflatoxin G2	331.0	189.2	313.2
Aflatoxin G1	329.0	200.2	243.2
Aflatoxin B2	315.1	287.2	243.2
Aflatoxin B1	312.9	285.2	241.2
Ochratoxin A	404.1	239.1	358.2

### Sample Preparation

About 2 g of sample (ground flowers or extract) was weighed out in a 50 ml centrifuge tube and then a 15 ml mixture of methanol: water (80:20) was added to the tube. The mixture was shaken vigorously for 60 min using the rotation shaker (70 rpm/min) and then centrifuged at 4,000 rpm for 15 min. About 6 ml of the supernatant was transferred to a new tube containing 20 ml of 2% Tween wash buffer in phosphate-buffered saline (PBS) and mixed well. A total of 26 ml of the diluted extract was passed through the IAC AOZON at a rate of 1 drop per second until air came through the column (it is important that the flow rate does not exceed 1 drop per second). The column was washed with three portions of 15 ml water (MS grade). Finally, the column was washed with 1 ml of methanol. That 1 ml is then diluted with 1 ml 0.2% formic acid. The sample solutions before injection in the system were filtered using a membrane filter (0.2 μm pore size).

### Standard Solutions and Calibration Curves

Working calibration solutions containing AflB1, AflB2, AflG1, and AflG2 were prepared from 0.1–5 μg/L in 50:50 water: methanol with 0.1% formic acid and for OchA 1–50μg/L in 50:50 water: methanol with 0.1% formic acid.

Due to the influence of the matrix on the result (difference in the slope of the curve between the standard prepared in methanol water 50:50 with 0.1% formic acid and the standard prepared in the matrix), the calibration curve was made in a matrix.

### Validation of the Method

The proposed method was validated according to the guidelines set by the International Conference of Harmonization for validation of analytical methods and the Directive 96/23/EC considering the performance of analytical methods and the interpretation of results (23, 24).

### Recovery Studies

To establish the accuracy of the proposed method, recovery experiments were carried out by adding the known amounts of the combined standard solution of AflB1, AflB2, AflG1, AflG2, and OchA to the decarboxylated oil.

### Experimental Design

At the beginning of the experiment, the concentration of AfB1, AfB2, AfG1, AfG2, and Och A in dry cannabis flowers (we use a variety of Herijuana) was determined. Then, AfB1, AfB2, AfG1, AfG2 (2 μg/kg), and Och A (20 μg/kg) were added to 250 g dried cannabis flowers, which were divided into three equal portions 2 h after adding of mycotoxins. The maceration was performed with 96% ethanol as a solvent, in a cold chamber (refrigerator at −20°C). The duration of the maceration was 30 min in total. Each portion of cannabis flowers was macerated in approximately 415 ml of 96% ethanol for 10 min separately (in total, 1.25 L of 96% ethanol was used for maceration). Stirring by stainless steel spoon was done on portion every 2 min. After maceration was completed, the macerated material (cannabis flowers) was manually squeezed with a stainless-steel strainer. The resulting macerate was filtered and collected in a pre-measured 1L beaker. The ethanol was evaporated on a hot plate. After evaporation of the ethanol, the obtained crude oil was decarboxylated by heating until the temperature of the crude extract reached 125–130°C. The experiment was performed three times (Batch No. RS0221/1, Batch No. RS0221/2, Batch No. RS0221/3).

## Results

### Validation of the Method

**Figure 4:**
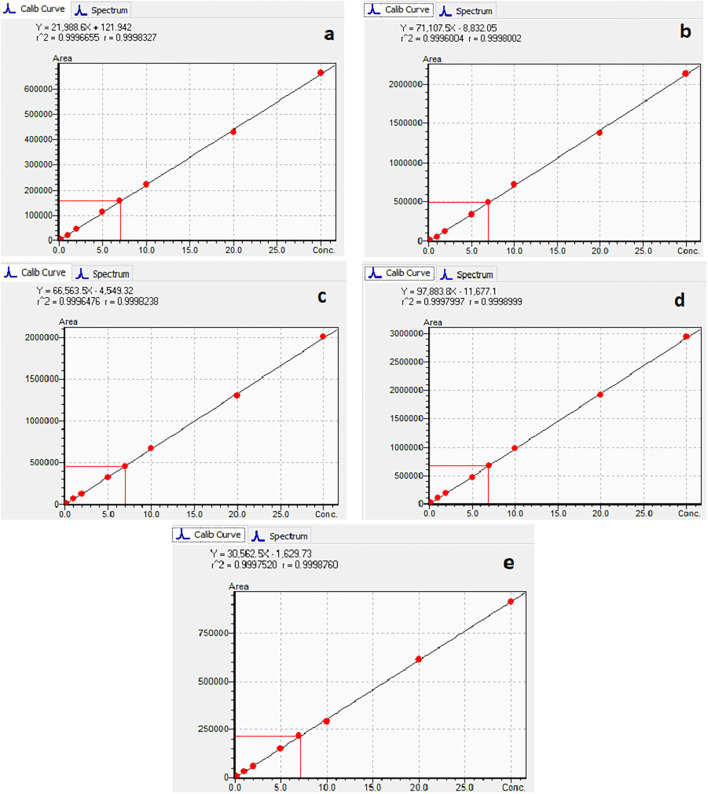
Calibration curves of AfG2 **(a)**, AfG1 **(b)**, AfB2 **(c)**, AfB1 **(d)**, OchA **(e)**.

The calibration characteristics and validation parameters of the proposed method are shown in Table 3 and Figure 4. Linearity of response was calculated as a ratio of peak areas of AflB1, AflB2, AflG1, AflG2, and OchA vs. concentration in the spiked samples in the concentration range of 0.1–5 μg/L in 50:50 water: methanol with 0.1% formic acid for AflB1, AflB2, AflG1, and AflG2 and OchA from 1–50 μg/L in 50:50 water: methanol with 0.1% formic acid. The correlation coefficient was >0.999 for all mycotoxins.

**Table 3:** Characteristics of the linear regression analysis.

	**AfG2**	**AfG1**	**AfB2**	**AfB1**	**OchA**
Linearity range (μg/L)	0.1–5	0.1–5	0.1–5	0.1–5	1 - 50
Determination coefficient (r2)	0.9998	0.9998	0.9998	0.9998	0.9998
CCα	4.32%	3.84%	4.55%	3.95%	3.87%

*CCα-Decision limit (max. allowed 5%)*.

The limit of detection (DL)/ limit of quantification (QL) was determined based on the formulas: DL = 3,3 x σ / S and QL = 10 x σ / S, where σ is the SD of the response and S is the slope of the calibration curve. The DL/QL for each mycotoxin separately is shown in Table 4.

**Table 4:** Precision and accuracy of the method.

**Concentration added (μg/L)**	**Measured concentration (μg/L)** ^**a**^			
	**AfG2**		**AfG1**	
	**Mean (μg/L) ± RSD (%)**	**Recovery (%)**	**Mean (μg/L) ± RSD (%)**	**Recovery (%)**
1.5	1.215 ± 0.96	81.0	1.214± 0.58	80.9
2.0	1.892 ± 0.82	94.6	1.940± 0.82	97.0
5.0	4.321 ± 0.78	86.42	4.696± 0.85	93.9
**Concentration added (μg/L)**	**Measured concentration (μg/L)** ^**a**^			
	**AfB2**		**AfB1**	
	**Mean (μg/L) ± RSD (%)**	**Recovery (%)**	**Mean (μg/L) ± RSD (%)**	**Recovery (%)**
1.5	1.228 ± 0.87	81.86	1.334± 0.71	88.93
2.0	1.927 ± 0.58	96.35	1.938± 0.49	96.9
5.0	4.283 ± 0.72	85.66	4.900± 0.57	98.0
**Concentration added (μg/L)**	**Measured concentration (μg/L)** ^**a**^			
	**OchA**			
	**Mean (μg/L) ± RSD (%)**	**Recovery (%)**		
15	14.09 ± 0.86	93.93		
20	20.93 ± 0.93	104.6		
50	50.59 ± 1.03	101.18		

a *Mean value of five determinations*.

Figure 4 shows the calibration curve of AfG2 (4-a), the calibration curve of AfG1 (4-b), the calibration curve of AfB2 (4-c), the calibration curve of AfB1 (4-d), and the calibration curve of OchA (4-e).

The results of precision, accuracy (recovery), and reproducibility (assay) of the method are shown in Table 5. They demonstrate good precision determined as the relative standard deviation (%RSD) accuracy, and reproducibility. RSD is the absolute value of the coefficient of variation and indicates whether the “regular” SD is a small or large quantity when compared to the mean for the data set.

**Table 5:** Limit of detection/Limit of quantification for AflB1, AflB2, AflG1, AflG2, and OchA.

**Mycotoxin**	**Limit of detection (μg/kg)**	**Limit of quantification (μg/kg)**
AflG2	0,023	0,069
AflG1	0,017	0,053
AflB2	0,034	0,105
AflB1	0,027	0,082
OchA	0,329	0,997

**Figure 5:**
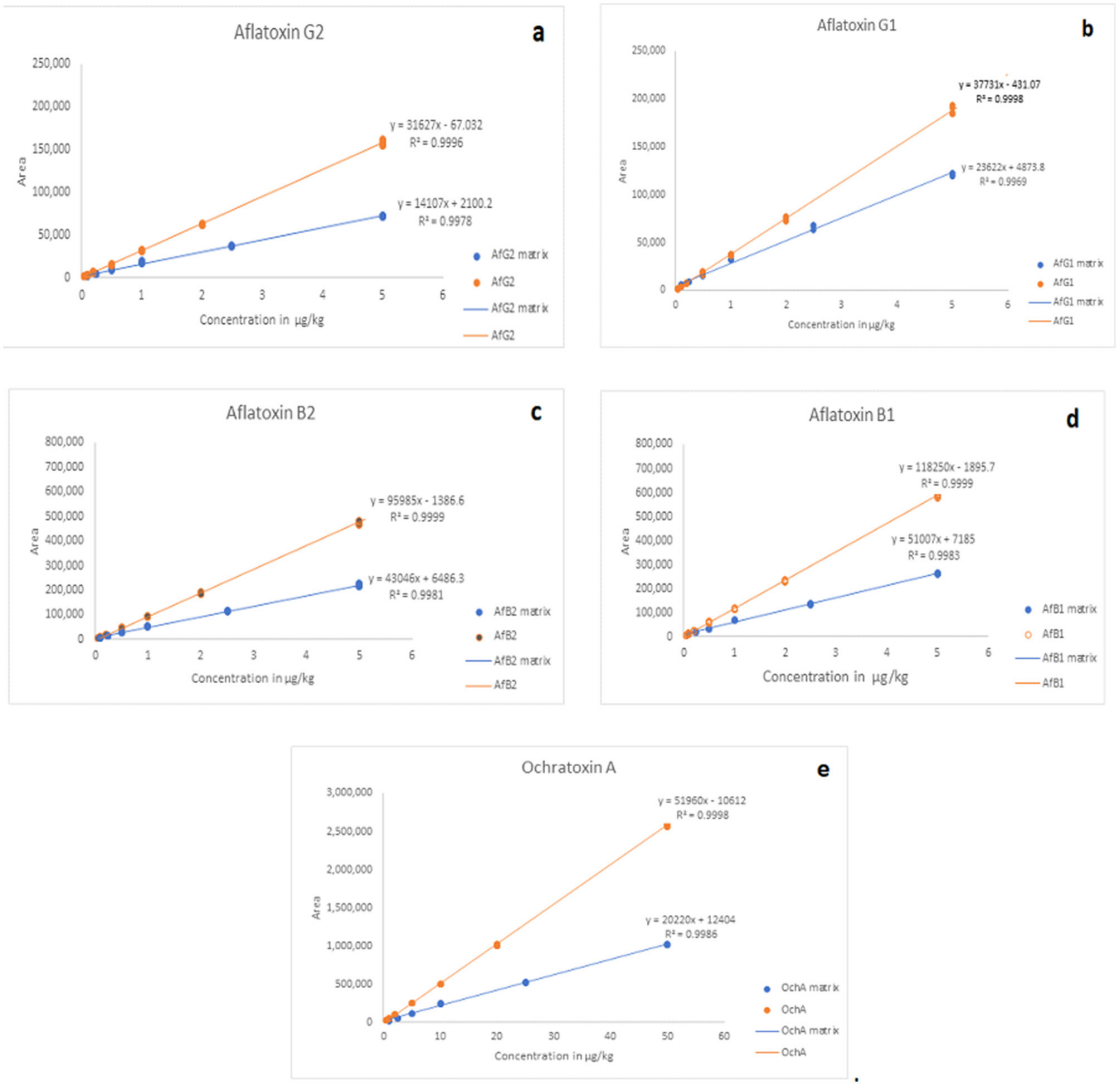
Comparison of matrix effects for AfG2 **(a)**, AfG1 **(b)**, AfB2 **(c)**, AfB1 **(d)**, and OchA **(e)**. Calibration curves of standard solutions of mycotoxins (red line) and calibration curves of standard solutions of mycotoxins in post immunoaffinity column matrix (blue line).

The matrix effect was evaluated using the post-extraction spike method, by adding standard solutions of mycotoxins into the matrix after extraction. Calibration curves of standard solutions of mycotoxins and spiked standard solutions of mycotoxins in the matrix were evaluated. It was shown that co-eluting compounds from the matrix affect the ionization efficiency and reproducibility in the ionization source (Figure 5). Therefore, all analyzes were evaluated using matrix calibration curves.

**Figure 6:**
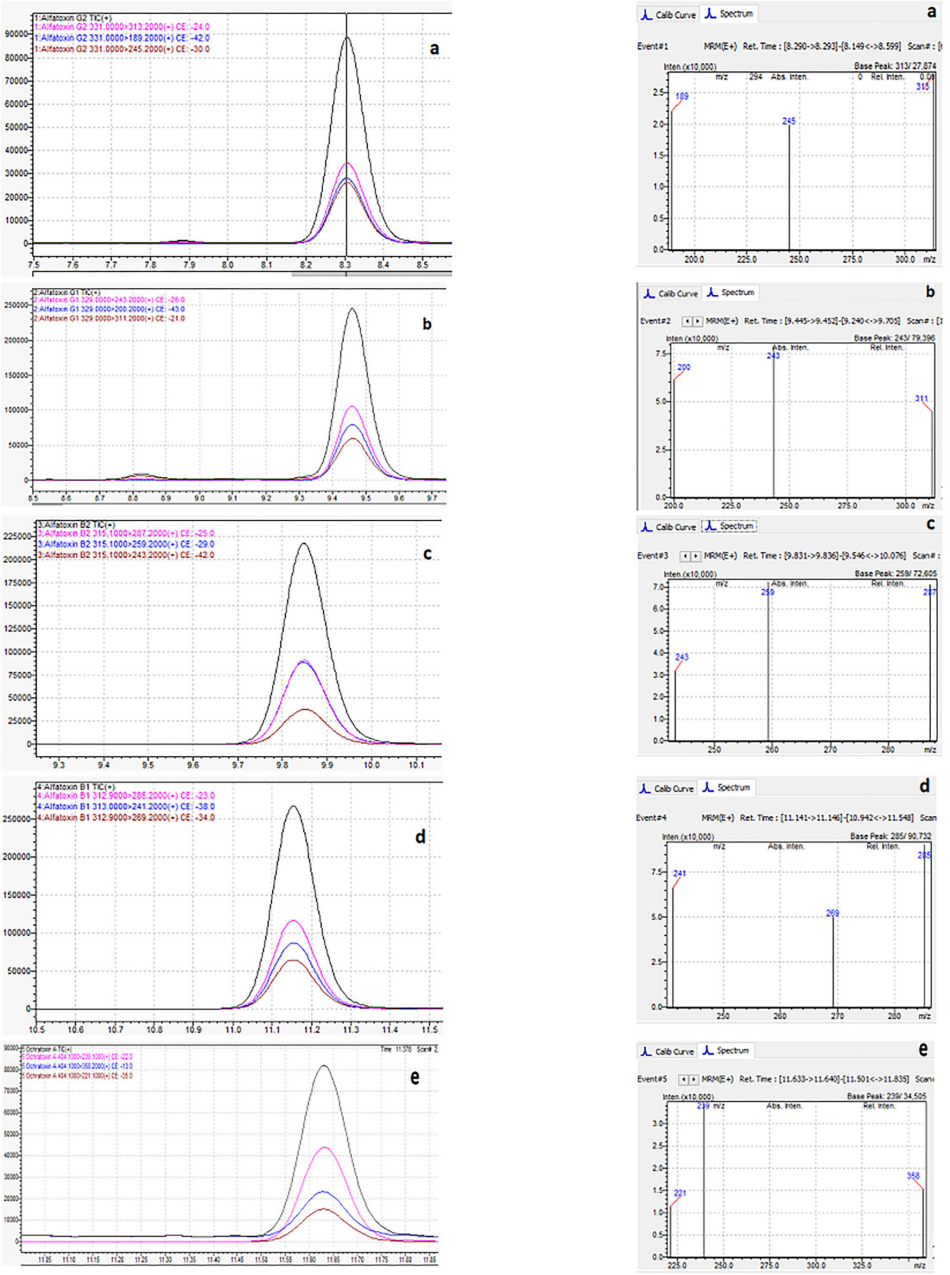
Typical chromatogram and analyte transitions of the of AfG2 **(a)**, AfG1 **(b)**, AfB2 **(c)**, AfB1 **(d)**, OchA **(e)**.

Figure 6 shows the typical chromatogram and analyte transitions of AfG2 (5-a), the typical chromatogram and analyte transitions of AfG1 (5-b), the typical chromatogram and analyte transitions of AfB2 (5-c), the typical chromatogram and analyte transitions of AfB1 (5-d), and the typical chromatogram and analyte transitions of OchA (5-e).

### Calculation of AflB1, AflB2, AflG1, AflG2, and OchA in Experimental Design

The concentration of aflatoxins and OchA were determined using the same LC/MS/MS analytical method in the starting material (dry flower) before spike and in obtained decarboxylated extract after extraction with ethanol 96%. The content of AflB1, AflB2, AflG1, AflG2, and OchA in microgram per kilogram of cannabis flower variety Herijuana (Table 6) and cannabis extracts (Table 7) obtained from cannabis flowers spiked with maximum residual level (MRL) allowed for aflatoxins (2 μg/kg) and (20 μg/kg) for OchA was calculated separately for each mycotoxin.

**Table 6:** The content of Aflatoxin B1, B2, G1, G2, and Ochratoxin A in cannabis dry flower variety Herijuana Batch No. 01012101 before spike.

	**Content of mycotoxins in cannabis dry (μg/kg)**
AfG2	Not detected
AfG1	Not detected
AfB2	Not detected
AfB1	Not detected
OchA	Not detected

**Table 7:** The content of Aflatoxin B1, B2, G1, G2 and Ochratoxin A in cannabis extract obtained from cannabis flowers spiked with maximum allowed residual level for aflatoxins and ochratoxin A.

	**RS0221/1**		**RS0221/2**		**RS0221/3**	
**AfG2**						
Added in flowers 2 μg/kg	Determined in extract (μg/kg)	(%)	Determined in extract (μg/kg)	(%)	Determined in extract (μg/kg)	(%)
	6.91	345.5	6.02	301.0	6.122	306.1
**AfG1**						
Added in flowers 2 μg/kg	Determined in extract (μg/kg)	(%)^*^	Determined in extract (μg/kg)	(%)^*^	Determined in extract (μg/kg)	(%)^*^
	6.90	345.0	6.32	316.0	6.42	321.0
**AfB2**						
Added in flowers 2 μg/kg	Determined in extract (μg/kg)	(%)^*^	Determined in extract (μg/kg)	(%)^*^	Determined in extract (μg/kg)	(%)^*^
	5.1	255.0	4.86	243.0	4.90	245.0
**AfB1**						
Added in flowers 2 μg/kg	Determined in extract (μg/kg)	(%)^*^	Determined in extract (μg/kg)	(%)^*^	Determined in extract (μg/kg)	(%)^*^
	5.06	253.0	4.59	229.5	4.62	231.0
**OchA**						
Added in flowers 20 μg/kg	Determined in extract (μg/kg)	(%)^*^	Determined in extract (μg/kg)	(%)^*^	Determined in extract (μg/kg)	(%)^*^
	141.6	708.0	119.9	599.5	121.9	609.5

* *Percentage from the maximum allowed concentration*.

The results of the assay in the not-spiked dry flowers (Table 6) show that there are no significant quantities of mycotoxins that could influence the validity of the experiment.

From the percent presented in Table 7, we can conclude that after evaporation of the solvent, aflatoxins, and OchA, remain in the final extract in amounts much higher than those in which the same are added.

**Figure 7:**
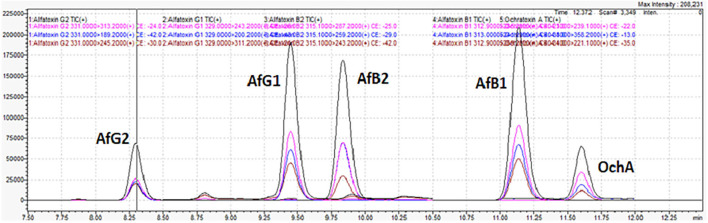
Chromatogram of cannabis extract obtained from cannabis floss spiked with MRL for aflatoxins (2 μg/kg) and ochratoxin A (20 μg/kg).

Figure 7 shows the chromatogram of cannabis extract obtained from cannabis flowers spiked with MRL for aflatoxins (2 μg/kg) and OchA (20 μg/kg).

## Discussion

Medical or recreational use of cannabis is now legal in more than 50 countries all over the world. With this emerging interest in cannabis, as governments regulate this field, there is an urgent need to understand possible contaminations and how this could impact human safety and health (25, 26). This is of particular concern for people who have compromised immune systems and use cannabis for medicinal purposes (26). In this study, we analyze the connection between cannabis cultivation and mycotoxins outcomes. Aflatoxins become especially problematic if the drying and storage of cannabis flowers are inappropriate (27).

Cannabis preparations are widespread products used to treat various painful and pathogenic conditions. The use of these preparations has been increasing in the last 10 years. Aflatoxins are a major source of disease, therefore, the extreme levels of aflatoxins in cannabis herbal preparations are of major concern. Post-harvest treatments to remove aflatoxins such as alkalization, ammonization, and heat or gamma radiation are not generally used and proposed. The M1 and M2 metabolic derivatives of AflB1 and AflB2 can be also excreted out through milk (9), and there could be a possibly harmful effect on breastfed infants.

Mycotoxins, especially aflatoxins, are extremely toxic secondary metabolites. They can cause disease and death in humans. Aflatoxins (Ph.Eur. 2.8.18) are limited to ≤ 2μg/kg for AfB1 and total aflatoxins (AflB1, AflB2, AflG1, AflG2) ≤ 4 μg/kg. OchA (Ph.Eur 2.8.22) is limited to ≤ 20μg/kg (5).

Standardized concentrated cannabinoid extracts, which are obtained from cannabis flowers, are used as a raw material for the preparation of cannabis products for human use. The extraction process is usually made by supercritical CO_2_ or ethanol (28, 29).

Ethanol, according to Ph.Eur., is classified as a Class 3 solvent with a low risk for acute or chronic toxicity in pharmaceutical manufacturing processes where the residual is <5,000 ppm (0.5%) (5). Ethanol is a very good solvent for the extraction of cannabinoids and terpenes and, at the same time, a very good solvent for mycotoxins. Extraction can be conducted under warm (room temperature) or cold conditions (supercooled ethanol extraction). The difference is that at room temperature generally more waxes and pigments are extracted which results in additional clarification steps than supercooled techniques. To extract the solutes from the flowers, ethanol must completely saturate the flowers. Therefore, a significant volume of ethanol is needed to perform the process with an efficiency rate of more than 90% (24). In our case, we use ethanol 96% in ratio flowers: ethanol = 1:5.

A novel sensitive, reproducible, rapid, and cost-effective LC/MS/MS method for the determination of aflatoxins and OchA was developed and validated. Recent trends of development in the methods of sample extraction, cleanup processes, detection technologies, quantitative methods, and the current research of fast and noninvasive detection methods were followed (8).

The flowers used for extraction were spiked with the maximum permitted level of mycotoxins according to Ph.Eur. (2.8.18 and 2.8.22) (5). Considering that AfB1 is limited to ≤ 2 μg/kg, means that every other mycotoxin (AfB2, AfG1, AfG2), individually, can be found in the flowers in the amount ≤ 2 μg/kg (assuming no presence of another aflatoxin), which means not to exceed the limit of total allowed mycotoxins ≤ 4 μg/kg. After evaporation of ethanol and decarboxylation process of crude, the content of mycotoxins was determined by the LC/MS/MS method.

The results obtained indicate that aflatoxins and OchA, although well soluble in ethanol, after evaporation of the solvent, remain in the final extract in an amount much higher than the amount in which we added them. If we consider that during the extraction the sample is concentrated (from 250 g of flowers, we obtained 16 g of extract), it means that the level of aflatoxins and OchA in the final extract is much higher than the maximum allowed level according to the Ph.Eur. (5), thus, posing a risk to human safety and health (determined percentage from the maximum allowed concentration: 301–345.5% for AfG2, 316–345% for AfG1, 243–255% for AfB2, 229.5–253% for AfB1, and 599.5–708% for OchA).

Therefore, it is very important that cannabis cultivation and post-harvest treatment including storage is performed under controlled conditions to avoid the formation of mycotoxins. In this way, human health can be protected and well preserved (25).

## Conclusion

Emerging interest in cannabis-based preparation for medicinal use has imposed an urgent need to understand possible contaminations, referring especially to the effect of aflatoxins which are extremely toxic secondary metabolites. These may pass from the contaminated plant material to the extract and have an impact on human safety and health. To demonstrate this, a novel sensitive, reproducible, rapid, and cost-effective LC/MS/MS method for the determination of aflatoxins and OchA in cannabis extracts was developed and validated. The results obtained from the testing indicate that aflatoxins and OchA, primarily added to the cannabis dried flowers, were also present in the obtained final extract, in amounts higher (m/m) than those in starting plant material. With this experiment, we have shown that aflatoxins as extremely toxic secondary metabolites, can reach critical values in cannabis extracts obtained from dry cannabis flowers with the maximum allowed quantity of aflatoxins. This can pose a great risk to consumers and their health especially to those with compromised immune systems.

## Data Availability Statement

The original contributions presented in the study are included in the article/supplementary material, further inquiries can be directed to the corresponding author/s.

## Author Contributions

All authors listed have made a substantial, direct and intellectual contribution to the work, and approved it for publication.

## Conflict of Interest

JE is employed by Diapharm GmbH & Co. KG, Münster, Germany. SS, ZK, and MM are employed by NYSK Holdings, Company for growing, extraction and producing of pharmaceutical dosage forms of medical cannabis, Skopje, North Macedonia. The remaining authors declare that the research was conducted in the absence of any commercial or financial relationships that could be construed as a potential conflict of interest.

## Publisher's Note

All claims expressed in this article are solely those of the authors and do not necessarily represent those of their affiliated organizations, or those of the publisher, the editors and the reviewers. Any product that may be evaluated in this article, or claim that may be made by its manufacturer, is not guaranteed or endorsed by the publisher.
